# Geographical migration and fitness dynamics of *Streptococcus pneumoniae*: a perspective from contrasting selective pressures

**DOI:** 10.3389/fmicb.2026.1952491

**Published:** 2026-10-12

**Authors:** Fan Zhang, Yulu Wang, Xuemei Li, Yun Li, Jianbo Xia

**Affiliations:** 1 Department of Clinical Laboratory Medicine, Maternal and Child Health Hospital of Hubei Province, Tongji Medical College, Huazhong University of Science and Technology, Wuhan, China; 2 School of Laboratory Medicine, Hubei University of Chinese Medicine, Wuhan, China; 3 Institute of Clinical Pharmacology, Peking University First Hospital, Beijing, China

**Keywords:** antibiotic pressure, antimicrobial resistance, phylogeography, *Streptococcus pneumoniae*, vaccine-driven selection

## Introduction

*Streptococcus pneumoniae* remains a leading cause of pneumonia, meningitis, and sepsis worldwide, imposing a substantial burden on global public health (Cliff et al., 2024; Mahtab et al., 2024). The pathogen's remarkable genetic plasticity, encompassing over 100 serotypes and more than 900 lineages, has enabled it to adapt rapidly to clinical interventions, including antimicrobial therapy and vaccination (Ganaie et al., 2025; Hanage et al., 2009). While pneumococcal conjugate vaccines (PCVs) have dramatically reduced vaccine-type disease in many high-income countries, serotype replacement and the expansion of resistant non-vaccine types have emerged as new challenges (Garcia Quesada et al., 2025; Bennett et al., 2025). Simultaneously, antimicrobial resistance (AMR) continues to escalate, particularly for macrolides and β-lactams, threatening the effectiveness of empirical treatment regimens (Zhou et al., 2022; Bruinsma et al., 2004).

Understanding the spatial spread and fitness dynamics of pneumococcal lineages is critical for designing effective surveillance and vaccination strategies. However, the complex interplay between human mobility, vaccine selection pressure, and antibiotic consumption remains poorly quantified, particularly in diverse epidemiological settings. Most phylogeographic studies have focused on high-income countries with established vaccination programs, leaving substantial gaps in our understanding of how these dynamics operate in regions with different selective pressures (Lo et al., 2019; Chaguza et al., 2016).

In this context, Belman and colleagues recently published a landmark study in Nature that integrates genomic data from 6,910 South African isolates with Meta human mobility data to reconstruct pneumococcal geographical migration and vaccine-driven fitness dynamics (Belman et al., 2024). Their study makes three pivotal contributions. First, it quantitatively demonstrates that pneumococci require approximately 50 years of transmission to become homogeneously mixed across South Africa—a finding that challenges intuitive assumptions about bacterial spread and reveals the deeply “entrenched” nature of endemic transmission. Second, by coupling Meta mobility data with phylogeographic models, the authors establish a quantitative link between individual-level behavior and pathogen macro-dispersal, showing that the “focal” nature of human movement drives slow spread. Third, they provide robust fitness estimates after PCV introduction: non-vaccine types (NVT) showed a relative fitness advantage of 1.68 (95% CI: 1.59–1.77) over vaccine types (VT), and penicillin resistance among NVT strains is gaining fitness, suggesting that vaccine-associated reductions in AMR may be transient (Belman et al., 2024).

While we greatly appreciate this high-quality work, we do not question the validity of their findings within the South African context. Rather, we argue that the extent to which their quantitative conclusions can inform policy in settings with fundamentally different selective pressures—such as China, where PCV coverage is < 5% but macrolide resistance exceeds 90%—remains an open empirical question. Our Chinese data do not invalidate Belman et al.'s findings but quantify how and to what extent pneumococcal fitness dynamics differ across contrasting epidemiological landscapes, thereby operationalizing the very context-dependence they themselves acknowledged (Belman et al., 2024, Limitations section).

In this opinion article, drawing on our 14-year nationwide surveillance in China (2007–2020, *n* = 1,312 isolates from 24 hospitals across 18 provinces) (Cui et al., 2026) and our phylogenomic analysis of the MDR clone CC271 (Zeng et al., 2023), we address a single overarching question: to what extent can phylogeographic and fitness estimates derived from one epidemiological setting inform surveillance and vaccine policy in another? We do not attempt to provide a definitive answer—such would require analyses across many more settings—but rather use the contrast between South Africa and China as an illustrative case study. Belman et al.'s study provides the reference point from a high-vaccine-coverage setting; our 14-year Chinese surveillance data provide the counterpoint from a low-vaccine-coverage, high-antibiotic-consumption setting.

## Geographical generalisability of phylogeographic findings

The South African dataset, while comprehensive, reflects the epidemiological landscape of an upper-middle-income African country with high PCV coverage (89% by 2022). The serotype distribution, clonal composition, and AMR profiles observed in South Africa differ substantially from those in other regions (Belman et al., 2024). Our 14-year surveillance in China demonstrates that serotypes 19F and 19A dominate, ST271 and ST320 are the predominant clones, and >90% of isolates are macrolide-resistant—features that are not mirrored in the South African data (Cui et al., 2026).

These differences are not merely descriptive but have profound implications for phylogeographic modeling. The “50-year” mixing time estimated for South Africa reflects the focal nature of human mobility in that specific geographic and demographic context. Countries with vastly different population densities, urban-rural distributions, transportation infrastructures, and mobility patterns may exhibit substantially different rates of pathogen dispersal. For instance, China's dense urban centers, extensive high-speed rail network, and high population mobility may facilitate more rapid inter-regional transmission than observed in South Africa. Whether the quantitative conclusions of Belman et al. can be extrapolated to countries with such differing characteristics remains an open question that requires validation through similar analyses in diverse settings.

## Contrasting drivers of MDR clone expansion

Belman et al. attribute fitness changes in South African pneumococci primarily to PCV introduction, which is plausible given the country's 89% coverage (Belman et al., 2024). However, in many parts of the world—especially low-and middle-income countries—pneumococcal vaccine uptake remains low (Clark et al., 2026). Our Chinese data show that while PCV13 serotypes account for 85.8% of pediatric isolates, actual vaccination coverage is only about 4.1% (Cui et al., 2026). This unique epidemiological context allowed us to investigate MDR clone dynamics largely unconfounded by vaccine pressure.

Our phylogenomic analysis of the MDR clone CC271 revealed a distinct evolutionary trajectory (Zeng et al., 2023). The dominant clone, 19F ST271-B, expanded rapidly during 1995–2000, coinciding with the widespread use of cephalosporins in China during the 1990s. This clone possesses unique PBP2x substitutions (M339F, M400T, Y595F) conferring significantly higher resistance to cephalosporins than other CC271 clones (Zeng et al., 2023). Thus, in settings without strong vaccine pressure, antibiotic selection appears to be the dominant driver of MDR clone expansion, although the potential contribution of vaccination—particularly given that PCV7 includes serotype 19F, the capsule type of ST271—cannot be entirely excluded. This contrast underscores that the relative importance of vaccine vs. antibiotic pressure is context-dependent and cannot be generalized from a single country.

The implications for public health policy are substantial. In regions with high antibiotic consumption but low vaccine coverage, antimicrobial stewardship may be as important as vaccination in controlling MDR clone expansion. Models that attribute fitness changes solely to vaccine pressure may underestimate the role of antibiotic selection and misguide intervention strategies.

## Global transmission of vaccine-escape clones across contrasting vaccination contexts

Belman et al. focus on NVT expansion within South Africa following PCV introduction. Our data add an important global dimension: the vaccine-escape clone 19A ST320, which emerged in the USA after PCV7 introduction in 2000, began expanding rapidly in China around 2001—despite PCV7 not being licensed in China until 2008 (Zeng et al., 2023). Phylogenetic analysis showed multiple subclades shared between China and other countries, suggesting possible international transmission (Zeng et al., 2023). Local antibiotic selection cannot be excluded as a driver of ST320 expansion in China, and that the vaccine-escape label is context-dependent.

This phenomenon raises the possibility that mass vaccination in one country can indirectly affect pathogen populations in unvaccinated countries through global transmission networks. The post-PCV7 emergence of 19A in the USA may have contributed to global dissemination that subsequently expanded in countries without local vaccine pressure. This finding has profound implications for global vaccine policy: the benefits of vaccination extend beyond national borders, but conversely, vaccine-driven clonal shifts in one region can introduce new threats to others.

This global dimension is absent from the South African analysis, which primarily considered within-country transmission. Future phylogeographic models must incorporate international transmission networks to fully capture the dynamics of vaccine-escape clones and inform coordinated global vaccination strategies.

## Sample representativeness and age-related biases

Although the South African dataset is large (*n* = 6,910), it has an age-related bias: 50% from children < 3 years old, only 17.5% from adults (Belman et al., 2024). Carriage rates, disease spectra, antibiotic exposure, and mobility behaviors differ markedly across age groups. Children are major reservoirs of pneumococcal carriage and transmission, but adults and the elderly bear the highest burden of invasive disease and mortality (Wahl et al., 2018; Bahrs et al., 2025).

Our Chinese study was specifically designed to stratify by age: children < 14 years, adults 15–64 years, and elderly ≥65 years (Cui et al., 2026). We found that β-lactam resistance in pediatric isolates was significantly higher than in adults and the elderly—an age-related difference that may be underestimated in the South African study. Higher resistance in children may be driven by greater antibiotic exposure (especially β-lactams and macrolides for respiratory infections), close contact in daycare settings, and clonal expansion of pediatric-adapted lineages like 19F ST271-B (Cui et al., 2026).

The age distribution of samples influences phylogeographic inferences because transmission networks and mobility patterns differ across age groups. Age-stratified analyses are essential for understanding the full dynamics of pneumococcal spread and fitness evolution. Future studies should ensure balanced age representation or incorporate age as a covariate in phylogeographic models.

## Long-term prediction of AMR fitness and data temporal mismatch

Belman et al. suggest that vaccine-associated declines in penicillin resistance may be temporary, as NVT-resistant strains are gaining fitness (Belman et al., 2024). In our Chinese context, we observed a different picture: MDR clones such as ST271-19F and ST320-19A remained dominant over 14 years with no statistically significant trend changes (Cui et al., 2026). This suggests that, in China, antibiotic pressure sustains MDR clone predominance independently of vaccine effects. The contrast underscores that the interplay between AMR and vaccination is highly dependent on local antibiotic usage, vaccine coverage, and clonal transmission networks.

Additionally, the Meta mobility data used by Belman et al. were collected during the COVID-19 pandemic (2020–2021), a period of unprecedented disruption to human movement (Belman et al., 2024). Using these data to infer transmission patterns from 2000 to 2014 introduces temporal mismatch. Although the authors partially adjusted for this with a parameter, residual confounding cannot be fully eliminated. Human mobility patterns changed substantially during the pandemic, with reduced long-distance travel and increased local movement. Whether these changes proportionally reflect pre-pandemic mobility patterns is uncertain.

Future studies should ideally use mobility data contemporaneous with the genomic samples or validate findings with multiple independent mobility datasets to ensure robustness.

## Discussion

The study by Belman et al. is methodologically groundbreaking and offers invaluable insights into pneumococcal spread and evolution in a high-vaccine-coverage setting (Belman et al., 2024). Their integration of genomic data with human mobility represents a significant advance in phylogeographic methodology, demonstrating how directly observed mobility data can be used to infer mechanistic transmission dynamics.

Our data from China—a setting with low vaccine coverage but high antibiotic consumption—suggest that antibiotic selection pressure can be a major driver of MDR clone expansion, and that global transmission of vaccine-escape clones can occur across settings with differing local vaccination pressure (Zeng et al., 2023; Cui et al., 2026). Also, the distinct epidemiology of pneumococcal infections in Chinese children underscores its context-dependent nature. A Beijing surveillance study showed *S. pneumoniae* ranked third among pediatric isolates, with 77.1% from children < 5 years and macrolide resistance >95% (Lyu et al., 2023). A pre-PCV13 IPD study identified serotypes 19F, 19A, 14, 23F, and 6B as predominant, with PCV13 coverage of 90.1%, yet 89.2% MDR, and MLST revealed antibiotic-driven clonal replacement without widespread vaccine pressure (Shi et al., 2019). These findings confirm that local antibiotic use and vaccination coverage profoundly shape pneumococcal epidemiology—factors distinct from South Africa (Belman et al., 2024). The high PCV13 coverage but extremely low uptake in China represents a missed prevention opportunity, emphasizing the need for context-specific surveillance and vaccine policies.

The heterogeneity of pneumococcal vaccination policies across countries adds another layer of context-dependence to pneumococcal epidemiology. While PCV7, PCV10, and PCV13 have been widely introduced in many high-income countries with routine childhood immunization programs, the timing of introduction, coverage rates, and use of PPV23 for older adults vary substantially (Bennett et al., 2025; Garcia Quesada et al., 2025). These policy differences directly shape serotype dynamics: in countries with high PCV coverage, vaccine-type serotypes have declined sharply, but non-vaccine serotypes—such as serotype 8 in England and Spain, and 19A in the United States post-PCV7—have expanded through serotype replacement (Lo et al., 2019; D'Aeth et al., 2025). In contrast, in countries like China where PCV uptake remains < 5%, vaccine-type serotypes (19F, 19A) continue to dominate without evidence of vaccine-driven replacement (Shi et al., 2019; Cui et al., 2026). This contrast underscores that serotype dynamics are not intrinsic to the pathogen but are profoundly shaped by local vaccination policies and coverage levels.

The strong and stable linkages between specific STs, serotypes, and GPSCs observed in our study suggest that CC271/GPSC1 has rarely undergone capsule switching in China over the past 14 years, as evidenced by the consistent 19F-ST271 and 19A-ST320 associations (Zeng et al., 2023; Cui et al., 2026). This stability implies that, if PCV13 coverage were increased, it would likely reduce the burden of these dominant MDR clones without immediate risk of vaccine escape through serotype replacement within the same genetic background. However, this conclusion warrants caution. Capsule switching, although a historically low-frequency event in this lineage, remains a theoretical possibility under sustained, long-term vaccine selection pressure (Croucher et al., 2014; Wyres et al., 2013). Continuous genomic surveillance will be essential to detect any emergence of capsule-switch variants should PCV13 uptake increase substantially in China.

The contrasting evidence from South Africa and China does not invalidate either dataset. Instead, it demonstrates that the drivers, pace, and trajectories of pneumococcal fitness dynamics are fundamentally context-dependent—a conclusion that Belman et al. themselves endorsed qualitatively, and that our Chinese data now provide complementary descriptive support. Future phylogeographic models must therefore incorporate local measures of antibiotic consumption, vaccination coverage, and age structure, and must be calibrated to the specific epidemiological context in which they are applied.

We acknowledge an evidentiary asymmetry between this article and Belman et al. provided quantitative fitness estimates with confidence intervals derived from formal phylogeographic modeling. By contrast, the Chinese evidence presented here is primarily descriptive, relying on dominant clones, resistance percentages, and the absence of a statistically significant temporal trend. These data are therefore not directly equivalent to formal fitness estimates. Rather, they provide complementary contextual evidence and should motivate future quantitative phylogeographic and fitness modeling using Chinese genomic, mobility, and antibiotic consumption data.
